# Cholera Infantum

**Published:** 1844-07

**Authors:** 


### ILLINOIS
### MEDICAL & SURGICAL JOURNAL.

VOL. I. JULY, 1844. NO. 4.

### CHOLERA INFANTUM.

THIS disease, so fatal in many portions of the American continent, is little known in Europe. Some writers upon the subject are indeed of opinion that it is exclusively American. Facts do not, however, appear entirely to carry out this view. A case is quoted by Dr. Horner,* from M. Billard, which was evidently, from the symptoms and post-mortem appearances, a true case of cholera infantum. Symptoms analogous to those essential to this disease, are enumerated by Dr. Hope,† as precursory to inflammation of the brain, or its membranes,—a frequent termination of cholera infantum. The diseases known as "infantile gastric remittent fever," "weaning brash," "worm fever," "bilious and mucous diarrhœa of children," all appear to have some symptoms and pathological relations in common with this disease. The essential points of distinction will be noticed when we come to speak of diagnosis.

*Symptoms.* Cholera infantum is generally ushered in by diarrhœa of several day's continuance. Violent vomiting and purging follow, with griping, and spasmodic pains similar to those of the cholera of adults. Fever generally supervenes, with "a quick and feeble, or irritated and corded pulse; seldom full, strong, or voluminous.‡ There is usually an unequal distribution of temperature, the head and epigastric region being preternaturally hot, while the extremities are cold. Determination to the brain is a frequent symptom. This is, in some instances, of an active character, as denoted by increased sensibility, intolerance of light, injection of the conjunctiva, restlessness, and sometimes convulsions. In other cases, and most frequently in the later stages, the

* American Journal of Medical Sciences, February, 1829.
†On Inflammation of the Brain. Tweedie's Library of Practical Medicine.
‡On Cholera Infantum. Dewees on Children, Philadelphia, 1836, p. 415.

cerebral symptoms are those of depression, such as deficient animation, languor, drowsiness, or stupor, the little patient sleeping with the eyes half closed. The dejections are, for the most part, white, thin, and frothy, sometimes tinged with blood, and sometimes containing small lumps of fœcal matter, covered with mucus. Occasionally the evacuations have a greenish tinge, less often are yellow or brown. They are either inodorous, or extremely offensive. According to Dr. James Jackson, the natural odor of fœcal evacuations is never present. The rapidity of emaciation is a most characteristic symptom, and Dr. Brainard has mentioned paleness of the surface, depression of the umbilicus, and a contraction of the skin of the abdomen into transverse wrinkles, or folds, as of constant occurrence in his practice.

If relief is not obtained, and death does not occur in a few days, the symptoms of collapse supervene. The patient becomes somnolent, is difficult to rouse, fainting fits occur, the countenance is sunken and pallid, and death may occur, with all the symptoms of hydrocephatus, or the little sufferer may linger for weeks under the most aggravated circumstances, and finally recover. After the violence of the attack has abated, the symptoms may change into those of simple chronic diarrhœa, and require the same treatment.

The *prognosis* is exceedingly difficult. The reappearance of bile in the stools, with diminished frequency and increased consistency; of moisture upon the skin; the cessation of the fits of vomiting; improved appetite, and increasing cheerfulness and attention to surrounding objects, are favorable symptoms. Dr. Dewees (loc. cit.) mentions two symptoms which he esteems as invariably fatal: "The appearance upon the chest of a crystalline eruption composed of a vast collection of minute vesicles, apparently produced by flirting an equal number of minute drops of boiling water," and the crawling of a *live* worm from the throat. Dr. Chapman mentions the occurrence of a pink margin in the stools as a fatal sign.

*Diagnosis.* Cholera infantum differs from the cholera of adults in several particulars. It is more distinctly febrile, is often gradual in its approach, and is apt to be protracted in its course, and assume a chronic form, circumstances seldom seen in the latter. (Eberle.*) It also exclusively attacks children between the ages of 2 and 24 months. It is distinguished from infantile

* Practice of Medicine, vol. ii. p. 304.

gastric remittent fever, by the occurrence of the latter in children over 2 years of age (Dr. Locock*); by the greater prominence of the febrile symptoms, by the regularity of the remissions, less frequent occurrence of cerebral symptoms and spasmodic pains, less rapid emaciation, and frequent constipation. From simple diarrhœa and dysentery, the gastric derangement, and the symptoms observed by Dr. Brainard, as mentioned above, will serve to distinguish it. The most difficult point of diagnosis, is between this disease and idiopathic affection of the membranes of the brain. The difficulty is enhanced by the fact that, in infants, we are obliged to rely upon the *objective* symptoms, the inability of the patient to communicate his sensations excluding us from deriving any information from the *subjective.* In cholera infantum, there is more apt to be tenderness upon pressure over the epigastric region and redness of the tongue, and the vomiting, though more obstinate, more readily yields to the proper remedies than in the case where the cerebral affection is primary. In the latter the evacuations are most frequently dark and offensive; in the former frequently colorless and odorless. Unless there has been diarrhœa previous to the violent attack, in cerebral affections, the bowels are mostly constipated; in cholera infantum, diarrhœa may be considered a constant symptom. For further discussion of this subject, I must refer the reader to the admirable treatise of Dr. Hope, above cited.

*Post-mortem Appearances.* The seat of the disease is located, by common consent, in the mucous coat of the stomach and bowels. Dr. Horner (loc. cit.) is of opinion, from numerous autopsies, that this affection is "rather a *follicular* than an *erythemoid* inflammation, that is, rather a disease of the innumerable mucous glands and follicles, extended from one end to the other of the alimentary canal, than a common vascular or erythemoid inflammation." In the stomach and small intestines, particularly the duodenum, dark livid patches have been observed. (Dewees' loc. cit.) According to the same author, the large intestines are seldom mnch affected; with this opinion Dr. Horner does not agree, for he remarks that ulcerations, though of rare occurrence, are more frequent in the large than the small intestines. The gall-bladder is sometimes distended, either with bile of a green color, or a fluid of a pale color like mucus; in one instance observed by Dr. Dewees, it was "filled with a fluid nearly as white as

* Tweedie's Lib. Pract. Med., vol. i. Art. Infantile Gastric Remittent Fever.

serum, and of little more consistency;" at other times it has been found flaccid, with the same kind of contents. The liver is generally enlarged, seldom altered in structure. Its enlargement is supposed to be dependent upon congestion, from obstruction of the portal circulation. The other viscera of the abdomen, and those of the chest, do not present any abnormal appearances. The brain in most cases appears in a state of congestion with red blood, and in protracted cases, effusions are found showing a co-existing inflammation of the arachnoid.

*Causes.* The remote causes of cholera infantum, are generally acknowledged to be high atmospheric temperature, vitiated air of cities, and the irritation of dentition. To these may be added surfeit, depraved nutrition, vitiation of the milk from ill health of the mother or other causes, weaning too early, inordinate purgation, and the abuse of calomel. As a frequent *proximate* cause of the disease, we are disposed to assign the presence of acid secretions. There are, we think, several good reasons for this presumption. When bile is present in the discharges, they almost invariably assume the "chopped spinage" appearance, which is referred to the presence of acid; a sour smell is frequent in the evacuations; the liver is torpid and the bile is absent, whose office it is to neutralize the excess of acid in the chyme; the mother's milk, which should be alkaline, is often acid; and the diarrhœa is checked more quickly and permanently, and, the function of the liver being restored, the natural appearance of the evacuations is more rapidly restored, by the use of alkaline remedies, than by any other means. "In some instances the discharges are so acrid as to excoriate the parts about the anus;" (Eberle loc. cit.); would they not then be sufficient cause for the follicular inflammation of the intestinal canal?

*Treatment.* If called to a patient affected with the precursory symptoms of cholera infantum, the indications are, to restore the function of the liver and skin; to free the alimentary canal of such ingesta as may be the immediate source of irritation, and to restrain within bounds the diarrhœa. If the evacuations contain no bilious matter, calomel with ipecac, each ¼ gr. may be given every two hours, until a fœcal or bilious evacuation has been obtained, which will generally be in six or eight hours. These minute doses answer the indications for their use, equally well with larger quantities, and are less apt to produce unpleasant results. When bile is present in the evacuations, Hyd. cum. creta, grs. ij. with creta prep. grs. iv. may be given every two, four, or six hours, according to circumstances, until the frequency and abnormal character of the discharges is obviated. If determination to the brain is evident, it becomes a matter of doubt whether the diarrhœa should be suddenly checked. The administration of astringent remedies under such circumstances, would, we think, be not only useless but injurious, and, in fact, their use is of doubtful propriety, except in later stages, where the diarrhœa is habitual, rather than a result of irritation. Remove the acid secretions by alkaline remedies, and restore the function of the liver, and the diarrhœa will of itself subside. If it be deemed advisable to give an alkali, without immediately causing a cessation of the discharges, the following will be found a good formula: ℞. soda sup. carb. ℈ij. aquæ menth. pip., aquæ font. aa fl℥j. M., of this a teaspoonful may be given, to a child 6 months old, every two hours until the discharges assume a natural tinge. The compounds of soda, potassa, and magnesia, form, with the acid secretions, purgative compounds, and keep the bowels in a soluble state, while the compounds with lime are without purgative action, and under their use the diarrhæa generally ceases suddenly. An exception to the condemnation of astringents, is found when blood appears in the stools. Under such circumstances, the use of plumbi acetas ⅛ gr. every two or four hours, will be found highly useful. This simple treatment, with proper regimen, will be found all-sufficient, in most of the diarrhœas of children, whether simple, or premonitory to an attack of cholera infantum.

The use of opium in any stage of cholera infantum we esteem worthy of the strongest condemnation. In the greater majority of cases, occurring under our own observation, where the brain or its members were affected, this drug in some form had been administered by parents or officious friends. Numerous professional gentlemen with whom I have conversed upon the subject have given their testimony to the same fact. We cannot forbear quoting the observations of Dr. Sigmond in regard to this point of practice, as we are persuaded that their importance is not sufficiently appreciated.* "To manage the use of opium, or other medicines, of the same class, adroitly, either in adults or in children, when it is our object to subdue nervous irritability is by no means an easy task. There are not many of the diseases to which infancy or childhood is subject in which you will find this drug at all necessary, and in

* Am. Journal, May, 1838, p. 182, from London Lancet.

by far the greater number it is altogether inadmissable; this most probably arises from the great predisposition that exists at that period to arterial acceleration and to cerebral affection. The only circumstances which imperatively call for its administration are very severe bowel complaints, which can by no other means be controlled, and also those alarming convulsions which occur at this period, and which sometimes threaten immediately to terminate existence; in such instances the necessity for quick relief, and the urgency of the case may demand from us, the having recourse to means which in themselves, under ordinary circumstances, would be objectionable. We must then administer very minute doses, must take care that if any unfavorable symptoms arise we have the means of checking their progress."

If violent vomiting and purgation come on, the treatment must be prompt and active. Internal remedies are seldom retained, so that it is necessary to tranquilise the stomach as promptly as possible by external applications. If the head is hot, the skin dry, the extremities cold, abstraction of blood from the temples by leeches, or other means, is an excellent preparatory to other treatment. Mustard applications to the feet, by the bath, or a large slice of bread moistened with hot water and sprinkled with mustard, the spice poultice over the abdomen are all to be put in requisition. "A teaspoonful of strong coffee, without sugar or milk, given every fifteen minutes," is highly recommended by Dewees. The same author speaks in the highest terms of "an injection of a gill of warm water, in which is dissolved a large teaspoonful of common salt; this is for a child a year old or upward, proportionably less for younger." This he recommends to be repeated *pro re nata* when the vomiting is severe until a copious fœcal or bilious evacuation is obtained. Cold applications should be kept upon the head as long as active determination to the brain is manifest, assisted by local abstraction of blood, or blisters behind the ears, as the symptoms may demand and the pulse admit. The state of the skin should be attended to and diaphoresis promoted by the warm bath, and in some instances where there is unequal distribution of temperature, this effect is assisted by blisters. "It is a fact not sufficiently known that without vesication, in certain conditions of the skin, diaphoresis will not take place." (Dewees loc. cit.)

If the above mentioned remedies do not arrest the vomiting, we have recourse to the powders of cal. and ipecac. aá gr. ¼. One of these may be administered every hour either by throwing it into the mouth, or mixing it with a small quantity of simple syrup, to which has been added its own bulk of a strong infusion of cardamon seed. These will generally procure a fœcal or bilious evacuation in a few hours; after which they may be given less frequently. When the irritability of the stomach has been allayed, the action of the powders may be assisted by a teaspoonful of melted butter, or castor oil, given every two hours until it operates; or by enemata containing a proportionable quantity of the same ingredients, with mucilage or starch. Active purges cannot but increase the irritation of the primæ viæ.

After the violence of the disease has abated, the treatment varies with the symptoms, and no precise rules can be given. If the diarrhœa continues, with light green discharges, the alkaline solution mentioned above, or the following formula, will be found useful: ℞ Hyd. cum. creta grs. xij. creta prep. ʒss div in chart. no. viij, of these one may be given every two, four, or six hours, according to circumstances. In some instances the diarrhœa becomes colliquative. It appears to be kept up by habit more than from any active cause of irritation. In several instances of this kind I was led to suppose that worms were the cause of its continuance, and with a view to their expulsion, administered the oil of turpentine. To my surprise the diarrhœa ceased, after a very few doses and not again returning, convalescence was rapid. In another instance I used the same remedy as a stimulant, in a case where there was evident irritation of the brain, with such general depression, that death was threatened simply from exhaustion. No evidences of effusion were discoverable but stupor and coma. Five drops of the oil of turpentine were given every four hours: the pulse rose in fulness, the passive diarrhœa was checked, the patient was more easily roused, and the system rallied to such an extent that I felt safe in applying a few leeches behind the ears. While the leeches were drawing every cerebral symptom disappeared, the diarrhœa did not return, digestion improved, and fresh air and wholesome diet completed the cure. I am inclined to think that the cases in which this remedy will be found most useful, are those in which the symptoms, from a resemblance to those of typhoid disease, lead us to suspect ulceration of the mucous membrane or the glands, which condition Dr. Horner's observations prove to exist. The value of this remedy in typhoid fever, as cited by Dr. Wood,* is well established, as also its general astringent effect upon mucous passages.†

In all stages of the disease it is essential to examine the gums and if swollen and tense in the situation of a tooth about to appear, free incisions are to be made *down to the tooth.* Upon this subject the following remarks of Dr. Hope are excellent: "The grand source of irritation resides not in the gum itself, but in the membrane immediately investing the tooth, which, formed out of cellular tissue, becoming progressively denser and tighter in proportion as it is more stretched by the enlarging tooth, eventually attains an exquisite degree of painful tension. When once this is fairly divided, it retracts so completely as ever after to be incapable of reuniting. It is the division of this membrane, therefore, which constitutes the great source of relief; if the superincumbent gum heal, any supposed induration of it is of little moment: the part has only to be divided again as often as it becomes inflamed." A simple incision, the whole length of the tooth, is to be practiced in the case of the incisors: in the molar teeth a crucial incision, so as to liberate the four corners.

During convalescence and after the violence of the disease is passed, the bowels should be kept in a soluble state, and digestion promoted by gentle tonics. These indications are both answered by the use of the aromatic syrup of rhubarb, of which a teaspoonful may be given twice or thrice daily, and if the laxative effect is not sufficient, more frequently.

*Regimen.* The importance of this part of the treatment cannot be too strongly insisted upon. If the child is still at the breast, a critical examination of the mother's milk should be made. This, from its liability to change in its constitution, from innumerable circumstances affecting the nurse, is without doubt one of the most pregnant causes of infantile diseases. The health of the nurse, her diet, her mental excitability, the effect of medicines, all, as causes of these changes, should be enquired into and regulated. The milk should be examined by chemical tests, and by the microscope, which has proved such a valuable means of investigating the state of the secretions in the hands of M. Donne.‡ This observer has shown the modes of ascertaining the abnormal

* U. S. Dispensatory Art. Oleum Terebinth.

† See Pereira's Mat. Med. Art. Oil of Turpentine, its use in Blenorrhœa.

‡ See Dictionnaire de Medecine Arts. "Lactation" and "Lait."

conditions of the milk, and indicated the means, in many instances, of correcting them. The milk should, in all cases, be alkaline in its reaction. The simple test with litmus paper, will indicate the presence of acid, which will generally be counteracted by the administration of carb. of soda to the nurse. Simple remedies such as this, will often prevent much mischief. It would be well if all practitioners were acquainted with the researches of M. Donne, further notice of which our limits, already transgressed, forbid. For the rest we shall not attempt any addition to the rules laid down in the treatises of Dewees, Eberle and others, with the single exception, that in most of these essays the use of farinacious articles, is, we may venture to suggest, carried too far. Those substances, lately proven by Liebig, incapable of being formed into the tissues and affording but few salts, cannot contribute to the production of healthy blood, and especially in the stages of weakness and convalescence, should not entirely take the place of nitrogenized substances. The avoidance of surfeit, fresh air, and wholesome and nutritious diet, are the best preventives to this fearful disease, and without their proper regulation, medication affords but temporary relief.

